# Cardiac Troponin I elevation after epileptic seizure

**DOI:** 10.1186/1471-2377-12-58

**Published:** 2012-07-17

**Authors:** Nicole Sieweke, Jens Allendörfer, Wolfgang Franzen, Andreas Feustel, Frank Reichenberger, Wolfgang Pabst, Heidrun Heidi Krämer, Manfred Kaps, Christian Tanislav

**Affiliations:** 1 Departments of Neurology, Justus Liebig University Giessen, Giessen, Germany; 2 Departments of Cardiology, Justus Liebig University Giessen, Giessen, Germany; 3 Departments of Nephrology, Justus Liebig University Giessen, Giessen, Germany; 4 Departments of Respiratory Medicine, Justus Liebig University Giessen, Giessen, Germany; 5 Departments of Institute for Biomedicine and Epidemiology, Justus Liebig University Giessen, Giessen, Germany; 6 Department of Neurology, Neurologische Klinik Bad Salzhausen, Justus Liebig University Giessen, Giessen, Germany; 7 Department of Neurology, Justus Liebig University, Giessen, Klinikstrasse 33, 35385, Giessen, Germany

**Keywords:** Seizure, Vascular risk profile, Epilepsy, Coronary syndrome, Cardiac troponin

## Abstract

**Background:**

Cardiac troponin-I (cTNI) is highly specific biomarker to prove myocardial damage, e.g. in acute coronary syndrome (ACS). However, it occurs in other conditions as well. We therefore analysed cTNI increase in patients after generalized convulsive seizure.

**Methods:**

Consecutive patients admitted with acute generalized convulsive seizure were included in case of cTNI measurement on admission. Among 898 selected cases, 53 patients were referred secondary to our department; in 845 cases cTNI measurements on admission were available. In case of multiple admissions (81 cases), only the first admission entered our analysis. In 17 patients elevated cTNI was determined due to ACS; in one patient a myocarditis was found. 5 patients suffered of relevant renal insufficiency. Finally 741 patients were included in the analysis. A cTNI cut-off level of ≥ 0.1 ng/ml was considered. Factors associated with a cTNI increase were analysed subsequently.

**Results:**

The mean age of the study population (n = 741) was 47.8 years (SD ± 18.6), 40.9% were female. In 50 patients (6.7%) a cTNI elevation of unknown origin was found; no obvious cardiac involvement could be detected in these patients who all remained asymptomatic. A vascular risk profile (including at least hypertension, hypercholesterolemia or diabetes) (OR = 3.62; CI: 1.59 to 8.21; *p* = 0.001) and elevated creatine kinase on admission (OR = 2.36; CI: 1.26 to 4.39; *p* = 0.002) were independent factors associated with cTNI release.

**Conclusion:**

cTNI release occurs in patients with generalized convulsive seizure with predominance in patients with vascular risk profile.

## Background

Cardio-specific biomarkers have been considered the most reliable factors for the diagnosis of myocardial infarction [1,2]. Especially cardiac troponin-I (cTNI) is highly specific for myocardial muscular tissue damage and is never expressed after skeletal musculature injury [3]. Unexplained cTNI increase occurring in different conditions, e.g. septic conditions, critical illness, endocrine or neurologic disorders led to the assumption of other mechanisms of cTNI release than commonly determined by myocardial ischemia in acute coronary syndrome (ACS) [4-11].

Approaching the pathophysiology of non-myocardial ischemic cTNI release, several investigations addressed disorders with involvement of the central nervous system in particular stroke and subarachnoidal haemorrhage [12-15]. Evidence on troponin elevation after seizures is based on limited data [16-20]. A better understanding on cTNI release in other conditions than an ACS is highly relevant with potential impact for the diagnostic and therapeutic management. Therefore in the present study we aimed to investigate the frequency of cTNI release after generalized convulsive seizure.

## Methods

### Patients and data acquisition

**Figure 1:**
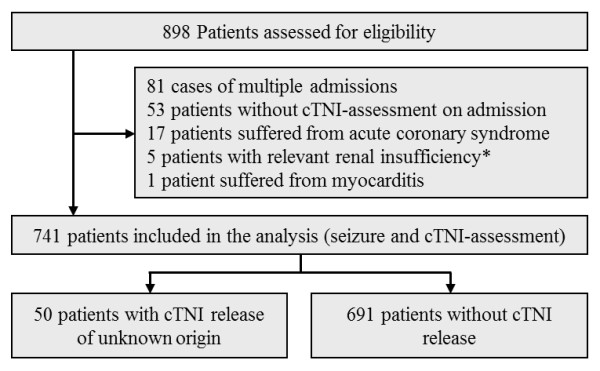
**Patients selection for eligibility (admission with generalized seizure and cardiac troponin I (cTNI) measurement).** Legend: * serum creatinine > 3.0 mg/dl.

Consecutive patients (n = 898) hospitalized in terms of an acute generalized convulsive seizure were identified from records relevant for hospital payment. The diagnosis of a generalized convulsive seizure was proven by reviewing the patients’ case files. The diagnosis of the generalized convulsive seizure was settled and documented in the payment records and patients’ case files according to the physician judgment. As the cTNI measurement on admission is implemented in our routine laboratory testing in a high percentage of patients, cTNI assessments were performed (n = 845). 53 patients were secondary referred to our department, thus cTNI measurements on initial admission were not available. In case of multiple admissions of an individual patient the first admission was considered; for these reasons 81 cases were excluded. In 17 patients an elevated cTNI was determined by an ACS; the diagnosis was established based on subsequent work-up. In 5 patients a progressed renal insufficiency (serum creatinine > 3.0 mg/dl) was evident. One patient was diagnosed with myocarditis. Other conditions known for causing a cTNI release including sepsis or rhabdomyolysis, concomitant pulmonary embolism, acute strokes or cerebral haemorrhage were not evident [4-10]. Finally, 741 patients with cTNI assessment on admission were included in the analysis (Figure 1).

The following parameters were systematically recorded: age, gender, known epilepsy, symptomatic epilepsy with type of lesion and localization, intake of anticonvulsants prior to admission, regular alcohol consumption prior to admission, previous myocardial infarction, known coronary heart disease (CAD) as diagnosed by coronary angiography, intake of beta-blockers, hypertension (if known and treated), hypercholesterolemia (if known and treated) and diabetes (if known and treated). If one of the conditions consisting of hypertension, hypercholesterolemia or diabetes were present, the patient was rated as having a vascular risk profile. The factor smoking could not be assessed due to inconsistent documentation. In patients with cTNI elevation the subsequent cardiac work-up was documented (coronary angiography if performed, echocardiography, ECG, 24 h-ECG, routine lab).

The study protocol was reviewed and approved by the local ethical committee of the Justus Liebig University, Giessen, Germany.

### Blood samples

For measurements of cTNI levels, the ADVIA Centaur® TnI-Ultra™ immunoassay (Bayer HealthCare, Tarrytown USA) was used. According to the local standards, a cTNI cut-off level of ≥ 0.1 ng/ml was considered a significant elevation; values of 0.1 ng/ml on admission were rated as increased when the second sample confirmed an elevation of ≥ 0.1 ng/ml.

The creatine kinase (CK) values were assessed using the CKNAC-kit for ADVIA Chemistry Systems and creatinine (Cr) values were measured by creatinin_2-kit for ADVIA Chemistry Systems (Bayer HealthCare, Tarrytown USA). A CK cut-off level of > 140U/l in females and > 174 U/l in males was considered as elevation. For Cr values cut-off levels of > 1.2 mg/dl in females and > 1.3 mg/dl in males were rated as significantly increased. Further, in all patients an estimated glomerular filtration rate (eGFR) was calculated according to the simplified MDRD equation: eGFR (ml/min/1,73 m^2^ body surface) = 186 x (serum creatinine)^-1.154^ x (age)^-0.203^ x 0.742 (if female) x 1.212 (if black) [21]. All routine laboratory tests were performed using certified methods.

### Statistical Evaluation

Normal distribution was verified by Kolmogorov-Smirnov’s one-sample test. Nonparametric data was analysed employing a Mann–Whitney U-test. For comparing relative frequencies, a Fisher’s exact test was used. Data significant on univariate analysis were entered into a logistic regression analysis based on a forward likelihood procedure. For the statistical analysis the SPSS Software (Statistical Package for Social Sciences) release 15.0 (SPSS Inc., Chicago, IL, USA) was used.

## Results

Among 741 patients who were included in the final analysis, 40.9% were female; the mean age was 47.8 years (SD ±17.5). In 50 patients (6.7%) a cTNI elevation was noted (Table 1). Symptoms related to an ACS were not present in any of these patients. Based on the recommendation of the cardiologists in 19 of these patients a coronary angiography was undertaken ruling out any findings suggestive of an ACS or relevant CAD. Serial ECGs, a 24 h-ECG and an echocardiography in all patients remained unremarkable. In all patients with cTNI release of unknown origin, a systematic retrospective review of ECG and 24 h-ECG examinations was undertaken, excluding any findings suggestive of acute myocardial ischemia. Echocardiography in all patients did not reveal any results indicating myocardial wall hypokinesia or dyskinesia; also an apical ballooning, the typical echocardiographic finding in a stress induced cardiomyopathy, also known as Takotsubo cardiomyopathy, could not be detected [22,23]. In 46 patients, maximum cTNI values of ≥ 0.2 ng/ml were found; in 4 patients a value of 0.1 ng/ml was not exceeded. In 40 patients the cTNI level decreased the day after admission, whereas in 10 patients a further increase could be observed (a cTNI value of 1.6 ng/ml was not exceeded).

**Table 1:** Gender specific comparison of CK and Cr values

	**cTNI elevation**	**cTNI negative**	***P***
**Female (n = 303)**	**n = 19**	**n = 284**	
CK values (U/l) on admission; median (range)	142 (39 – 7705)	137 (12 – 1792)	0.07
Elevated CK values on admission	10 (52.6%)	105 (37.0%)	0.22
Further CK increase after admission	9 (47.4%)	69 (24.3%)	0.05
Cr values (mg/dl) on admission, median (range)	1.0 (0.7 – 1.6)	1.0 (0.5 – 2.6)	0.35
Elevated Cr values on admission	5 (26.3%)	29 (10.2%)	0.05
**Male (n = 438)**	**n = 31**	**n = 407**	
CK values (U/l) on admission; median (range)	167 (29 – 13547)	145 (21 – 42143)	0.46
Elevated CK values on admission	17 (54.8%)	171 (42.0%)	0.18
Further CK increase after admission	15 (48.4%)	94 (23.1%)	0.004
Cr values (mg/dl) on admission, median (range)	1.2 (0.1 – 1.9)	1.1 (0.5 – 2.8)	0.36
Elevated Cr values on admission	11 (35.5%)	40 (9.8%)	<0.001

cTNI refers to serum cardiac troponin I.

CK refers to serum creatine kinase.

Cr refers to serum creatinine.

CK cut-off level > 140U/l in females and > 174 U/l in males.

Cr cut-off level > 1.2 mg/dl in females and > 1.3 mg/dl in males.

In 303 patients (40.9%), an elevated CK on admission was evident; a further increase of CK after the initial evaluation was observed in 187 (25.2%) participants. Elevated Cr values on admission were found in 85 patients (11.5%). There was no difference in the calculated eGFR values in the group with cTNI elevation versus without (Table 2). As for CK and Cr normal ranges are sex dependent, absolute values for males and females are separately reported in Table 1.

**Table 2:** Relationship between selected parameters and cTNI elevation in patients with epileptic seizure

	**Total cohort (n = 741)**	**cTNI elevation(n = 50)**	**cTNI negative(n = 691)**	P*	***P***^†^
Age (years); median (range)	47.8 (17.5 –89.9)	61.9 (21.6 – 88.1)	46.8 (17.5 – 89.9)	<0.001	0.07
Gender				0.77	
Female	303 (40.9%)	19 (38%)	284 (41.1%)		
Male	438 (59.1%)	31 (62%)	407 (58.9%)		
CK elevated on admission	303 (40.9%)	27(54%)	276 (39.9%)	<0.001	**0.002**
Further subsequent CK increase after admission	187 (25.2%)	24 (48%)	163 (23.6%)	<0.001	
Cr elevated on admission	85 (11.5%)	16 (32%)	69 (10%)	0.05	0.14
eGFR (ml/min/1,73 m^2^ body surface), median (range)	76.9 (43.8 – 162.8)	76.1 (45.9 – 162.8)	76.9 (43.8 – 159.3)	0.40	
Hypertension	155 (20.9%)	16 (32%)	139 (20.1%)	0.07	
Hypercholesterolemia	117 (15.8%)	12 (24%)	105 (15.2%)	0.11	
Diabetes	75 (10.1%)	9 (12%)	66 (9.6%)	0.08	
Intake of Beta-blocker	92(12.4%)	15(30%)	77 (11.1%)	<0.001	0.63
Known CAD	96 (13%)	18 (36%)	78 (11.3%)	<0.001	0.56
Vascular risk profile	193 (33.9%)	32(64%)	161(23.3%)	<0.001	**0.001**
Regular alcohol consumption	222 (30%)	17 (34%)	205 (29.7%)	0.52	
Known epilepsy	251 (33.9%)	25 (50%)	226 (32.7%)	0.02	0.18
Symptomatic epilepsy	249 (33.6%)	23 (46%)	226 (32.7%)	0.06	0.52
Intake anticonvulsants	231 (31.2%)	23 (46%)	208 (30.1%)	0.02	0.34

*P value calculated in the univariate analysis.

^†^*P* value calculated in the logistical regression.

CK refers to serum creatine kinase.

Cr refers to serum creatinine.

CK cut-off level > 140U/l in females and > 174 U/l in males.

Cr cut-off level > 1.2 mg/dl in females and > 1.3 mg/dl in males.

eGFR refers to estimated glomerular filtration rate.

cTNI release was associated with age, elevated CK and Cr on admission, intake of beta-blockers, known CAD, vascular risk profile, known epilepsy and intake of anticonvulsants (Table 2). Entered into a logistical regression analysis, a vascular risk profile (p = 0.001; OR: 3.62; CI: 1.59-8.21) and elevated CK values on admission (p = 0.002; OR: 2.36; CI: 1.26-4.39) were independently associated with cTNI release. In patients with a symptomatic epilepsy a cTNI release did not correlate with the location of the corresponding lesion (left sided lesions: 13/23 vs. 132/226; right sided lesions: 15/23 vs. 141/226, p = 0.99) or bilateral lesions (5/23 vs. 47/226, p = 0.99).

## Discussion

The primary finding of the present study is a cTNI elevation of unknown origin in a remarkable percentage of patients (6.7%) presenting with acute generalized convulsive seizures. A vascular risk profile - defined as the presence of at least one of the risk factors hypertension, diabetes or hypercholesterolemia - was identified as the main independent associated factor.

The pathophysiology behind the cTNI release of unknown origin in patients with different disorders is controversial. Some authors support the hypothesis of a “reversible ischemia model” due to a temporarily reduced coronary blood flow [7,13,20,24]. Others groups propose an autonomic involvement resulting from alterations of the central nervous system, predominantly causing a sympathetic overactivity [12,14]. Taking into account the short interval of global ischemia during a generalized convulsive seizure together with a pre-existing cardiac microvascular disturbance as a result of vascular burden, our results support the mechanism of a temporary cardiac ischemia. This mechanism explains potentially the regular findings in the subsequent cardiac work-up and the lack of cardiac clinical signs.

The presence of vascular risk factors consisting of hypertension and/or hypercholesterolemia and/or diabetes was the main factor associated with a cTNI elevation. Reviewing the literature the vascular burden is substantially represented in cohorts investigating a cTNI elevation without obvious cardiac involvement. The distribution of risk factors in these studies complies well with our findings. In stroke cohorts, a vascular burden per se could be assumed [25,26]. In studies investigating a cTNI elevation in critical illness in a high percentage of patients vascular risk factors were evident: diabetes 33%, hypertension 53% and hypercholesterolemia 37% [24]. Even analyses within heterogeneous cohorts demonstrated a considerable vascular burden [7]. Our findings might also support the hypothesis of vascular risk profile being a prerequisite for cTNI elevation in other conditions then ACS. In this context there is no predominating risk factor; the hypertension seems to possess the strongest association with increased cTNI levels.

There is evidence supporting the hypothesis that CK levels directly correlate with seizure duration [27,28]. Increased CK levels could therefore be interpreted as a result of extended phases of global ischemia. In our study, patients with cTNI release presented more frequently with elevated CK values. This is in line with findings depicted by Eskandarian and co-workers, who demonstrated on average higher troponin levels after complicated seizers as indicated by different heart circulatory parameters [16]. In this context, supposing larger intervals of general ischemia in case of “complicated” seizures, this might also support the mechanism of a temporary ischemia behind cTNI elevation.

The approach to localize a specific brain region possibly generating cTNI release is controversial [29,30]. The heterogeneity of lesions in our study does not allow a more precise analysis. However, specific hemisphere vulnerability for inducing a cTNI elevation was not evident.

The implementation of cTNI measurement in routine laboratory testing on admission enabled the acquisition of a large series of patients with cTNI assessment after an acute generalized convulsive seizure. Missing patients are unlikely because the data base used is also the basis for hospital payment. Similar to previous investigations, a detailed evaluation of the renal function is not available in our study. As some investigators identified a reduced glomerular filtration rate as potentially responsible for increased troponin levels, in this context detailed evaluation of the renal function appears mandatory. In our study increased creatinine levels, allowing certain estimation of the renal function were not associated with a cTNI increase [31]. Concerning the renal function, there were no relevant differences in the estimated glomerular filtration rate values between patients with cTNI elevation versus without. This suggests the observed cTNI elevation did not result from an impaired renal function.

## Methodological limitations of the study

Due to its retrospective design, the main limitation of the present study is the inability to reliably estimate the duration of the seizure in an individual patient. As general ischemia seems to be defining factor for the development of seizure-related cTNI elevation, the duration of the event is important [16]. Hence, this aspect needs verification in further examinations especially as elevated CK-levels indicating extended seizures are associated with cTNI elevation [27,28].

Blood samples for cTNI measurements were routinely collected on admission. However, the time between the event and the admission was not regularly documented in the patients’ case records. Therefore, the retrospective data acquisition does not allow a precise determination of these time intervals. As the period from seizure to admission might influence the cTNI-level, this aspect needs to be considered as potential bias interpreting the current results.

A further limitation of the present study is the heterogeneous cardiac work-up in patients with cTNI release. However, in all patients an ACS as a cause of cTNI elevation was sufficiently ruled out. The systematic retrospective review of relevant cardiological examinations (ECG, 24 h-ECG and echocardiography) did not reveal findings suggestive of acute myocardial ischemia or even a Takotsubo cardiomyopathy.

Patients with generalized seizures were identified from a database collecting relevant data for hospital payment. The diagnosis is therefore based on the treating physicians’ judgment. To minimize bias caused by transcription errors, all patients’ files were intensively reviewed in order to prove the qualifying event.

## Conclusion

A cTNI release occurs after generalized convulsive seizure with predominance in patients with vascular risk factors. Elevated CK values, as a potential marker for extended seizures, are associated with this phenomenon. Since cTNI is highly relevant for the diagnosis of myocardial tissue damage, further approaches investigating the mechanisms cTNI release are promptly required.

## Ethical approval

The study protocol was reviewed and approved by the local ethical committee of the Justus Liebig University Giessen.

## Abbreviations

cTNI: Cardiac troponin-I; ACS: Acute coronary syndrome; ECG: Electrocardiogram; CK: Creatine kinase; Cr: Creatinine; eGFR: Estimated glomerular filtration rate; SPSS: Statistical Package for Social Sciences.

## Competing interests

The authors declare they have no financial or non-financial competing interests.

## Authors’ contributions

NS, CT and JA carried out the data collection and drafted the manuscript. WP and CT performed the statistical analyses. All authors were involved in the analysis and interpretation of the results. All authors revised the manuscript critically for important intellectual content and helped to draft the manuscript. All authors read and approved the final version of the manuscript.

## Disclosures

All authors report no disclosures.

## Source of Funding

Study sponsorship: none.

## Pre-publication history

The pre-publication history for this paper can be accessed here:

http://www.biomedcentral.com/1471-2377/12/58/prepub
